# Human genetic influences on early B cell development

**DOI:** 10.70962/jhi.20250042

**Published:** 2025-06-25

**Authors:** Anna-Lena Neehus, Neil Romberg, Vijay G. Sankaran

**Affiliations:** 1 Division of Hematology/Oncology, Boston Children’s Hospital, Harvard Medical School, Boston, MA, USA; 2 Department of Pediatric Oncology, Dana-Farber Cancer Institute, Harvard Medical School, Boston, MA, USA; 3 Howard Hughes Medical Institute, Boston, MA, USA; 4 Broad Institute of MIT and Harvard, Cambridge, MA, USA; 5 Division of Immunology and Allergy, Children’s Hospital of Philadelphia, Philadelphia, PA, USA; 6 Department of Pediatrics, Perelman School of Medicine, Philadelphia, PA, USA; 7 Institute for Immunology and Immune Health, Perelman School of Medicine, University of Pennsylvania, Philadelphia, PA, USA; 8 Harvard Stem Cell Institute, Cambridge, MA, USA

## Abstract

Early B cells develop centrally and then migrate peripherally to mediate the essential immune functions of antigen presentation, immune regulation, and immunoglobulin production. B cell development is tightly regulated, ensuring the generation of distinctive B cell clones, each carrying a fixed B cell receptor and therefore antigen specificity. Defects in B cell development can underlie a variety of clinical phenotypes, including immunodeficiency, autoimmunity, and B cell leukemia. The study of human genetic variation has enabled the discovery of critical pathways for immune cell development, differentiation, and generation of immune repertoire diversity. Here, we focus on the complete allelic spectrum impacting central B cell development, including rare and common genetic variation, to shed light on unique and shared mechanisms underlying predisposition to B cell lymphopenia, autoimmunity, and leukemia.

## Teaser

This review explores how genetic variation impacts early B cell development, highlighting key genes and pathways involved in lineage commitment, V(D)J recombination, and B cell maturation. It discusses inherited rare variants and more common polymorphisms that predispose to B cell–related immune disorders and malignancies.

## Introduction

Antibodies are powerful effectors of adaptive immunity that surveil the interstitial spaces between cells. Antibodies are produced exclusively by B lymphocytes. Unlike T lymphocytes, which rely upon non-hematopoietic thymic accessory cells to develop and tolerize, each B lymphocyte precursor is intrinsically capable of sensing if its B cell receptor (BCR) is functional and if it recognizes self. B cells achieve this feat by passing through a self-imposed, stepwise series of quality control events that rely upon an elaborate and finely tuned network of recombination and cell signaling molecules.

Human genetic variation that perturbs these systems sheds light on which pathways are essential for early B cell development and self-determination. This review aims to first provide an overview of idealized B cell development, which purges all autoreactive clones before they can emigrate to the periphery. In reality, even the fittest among us have autoreactive and polyreactive naïve B cells in circulation that potentially provide a net health benefit (1, 2). In its second section, we will lay out a complete allelic spectrum of particularly rare and common genetic variants that impact early B cell development. We will also highlight the consequences of these distinct and shared genetic architectures that manifest as susceptibilities to infections, autoimmune diseases, and childhood B cell leukemia.

## Early B cell development

**Figure 1.**
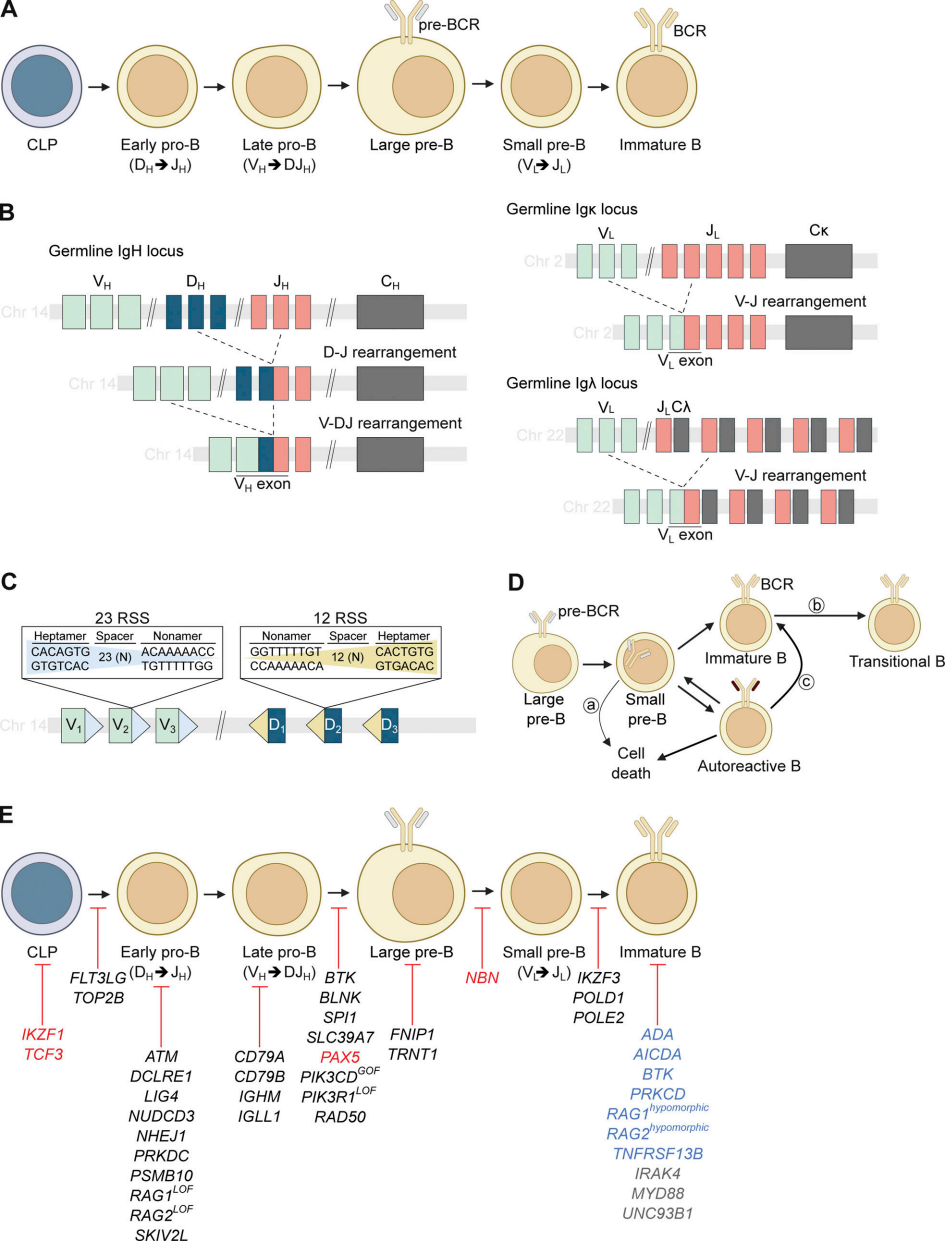
**Mechanisms of B cell development. (A)** Schematic representation of central B cell development occurring in the bone marrow. Key intermediated cellular stages are indicated as well as germline V(D)J recombination steps at the heavy (_H_) or light (_L_) chain locus. **(B)** Schematic representation of V(D)J recombination in the IgH (left) and light (right) chain loci. The germline IgH locus contains multiple V, D, and J gene segments upstream of the constant (C) regions. During recombination, one D gene segment is rearranged to join one J segment (D-J rearrangement), following by joining of one V gene segment to the previously rearranged DJ segment (V-DJ rearrangement). For simplicity only few V, D, and J segments and one C region are depicted. **(C)** Representation of 23 and 12 RSS sites flanking the IgH gene segments. **(D)** Schematic showing BCR-dependent maturation of small pre-B cells. Small pre-B cells internalize the pre-BCR and can (a) be subjected to deletion/energy, if no functional L-chain rearrangement occurs, (b) mature into non-self-reactive immature B cells, or (c) mature to autoreactive B cells that can undergo receptor editing, which can result in the expression of a non-self-reactive light chain and maturation into immature B cells. **(E)** IEIs affecting central B cell development and tolerance. Genetic variants leading to disrupted B cell development are indicated in black. Genes in which variants can underlie B cell deficiency and B-ALL predisposition are indicated in red. Variants affecting central B cell tolerance are indicated in blue. Genes affecting central B cell tolerance but without presentation of autoimmune phenotypes are indicated in grey. RSS, recombination signal sequence.

Like all blood cells, B cells develop from hematopoietic stem cells (HSCs) through a series of well-defined stages: HSCs yield lymphoid-primed multipotent progenitors and common lymphoid progenitors (CLPs), which then give rise to progenitor (pro-B), precursor (pre-B) (large and small pre-B), and finally immature B cells (Fig. 1 A) (3). While traditionally each of these developmental stages was thought to represent distinct cell states, more recent studies have revealed considerable heterogeneity at each stage (4). The developmental transition between pro- and pre-B stages is characterized by the sequential, stochastic somatic rearrangement of the gene segments encoding the variable (V), diversity (D), and joining (J) elements of the immunoglobulin heavy (IgH) chain (5) (Fig. 1 B). The process of V(D)J recombination is tightly controlled and regulated during lymphocyte development. In developing B cells, lineage-specific transcription factors such as E2A, EBF1, and PAX5 are recruited to enhancers within the immunoglobulin loci, thereby leading to transcriptional activity and chromatin accessibility, which allows for the binding of the lymphocyte-specific recombination-activating gene (RAG) enzymes at distinct recombination signal sequence sites flanking the V, D, and J segments (Fig. 1 C) (6, 7). The stochastic joining of one V, one D, and one J segment generates extensive combinatorial diversity that is enhanced by a suite of other excision and repair enzymes that add an arbitrary number of palindromic (p) and non-templated (n) nucleotides between gene segment joints (8). Only one third of p and n nucleotide additions maintain an open reading frame, meaning ∼66% of rearrangements fail (9, 10). This poses an important problem since each *IGH* locus can only be rearranged once because all unused D segments are episomally expelled from chromosome 14 during the primary recombination event. Hence, each B cell only has two chances to generate an in-frame heavy chain with an overall success rate of ∼55%. To identify and purge the remaining 45% of B cell clones that fail to generate heavy chains, pre-B cells express a pre-BCR comprised of a heavy chain and a surrogate light chain. All cells expressing intact pre-BCRs generate an “all or none” survival signal wholly dependent on the presence of an in-frame heavy chain and completely independent of its antigen specificity (11, 12).

Surviving pre-B cells downregulate their pre-BCR and initiate recombination of the immunoglobulin κ and λ light chain gene loci, located on chromosome 2 and 22, respectively (Fig. 1 B). As with heavy chain loci, light chain recombination generates diversity through random joining of V and J gene segments and the addition of arbitrary p and n nucleotides. As heavy and light chain loci recombination events are completely independent, the pairing of heavy and light immunoglobulin chains is essentially random, breeding an additional layer of “chain pairing” diversity. Although each B cell is theoretically capable of producing two different IgH chains (one from each chromosome) and four IgL chains (one κ and one λ per chromosome) at once, most B cells express a fixed pair with a defined ligand-binding specificity (13). Within the IgH locus, feedback regulation from a µ heavy chain produced from productive VDJ rearrangement on one allele suppresses V-to-DJ rearrangement on the other allele of the cell, thus resulting in “allelic exclusion.” This process ensures that a given B cell expresses a productive heavy chain from only one of its two alleles. B cells are isotypically excluded in that they only express κ or λ chains, but not both. This isotypic exclusion reflects a defined developmental sequence of light chain rearrangement with κ preceding λ. If the rearrangement between the κ V and J gene segments produces a nonfunctional VJ product, the locus undergoes segmental deletion through a rearrangement with the κ-deleting element, which is located downstream of the *IGCK* gene, and the developing B cell continues light chain rearrangement as the λ locus (14).

## Establishment of central B cell tolerance

The essentially random nature of V(D)J recombination creates a clonally heterogenous pool of developing B cells that can be practically divided into three groups based upon BCR reactivities to self-antigens (Fig. 1 D). The first group is small pre-B cells expressing BCRs incorporating out-of-frame IgL. Unable to generate any BCR signal, they must immediately reattempt light chain rearrangement or be clonally deleted. Unlike the heavy chain loci, which expel the D segment during primary recombination, unused upstream V and downstream J segments remain available for sequential secondary recombination attempts until these are exhausted. When IgL and IgH chain pairing is productive, the immature B cell will receive tonic BCR signals, irrespective of its BCR specificity. B cells expressing in-frame IgL chains that do not recognize self-antigens form the second group of developing B cells. These innocuous clones generate weak BCR signals and egress to surveil the periphery for their cognate antigen (15). The third and by far largest fraction of immature B cells are those that recognize self-antigens (1). Ligation of the BCR of the autoreactive clones will lead to activation of BCR-associated kinases that will ultimately promote the re-expression of RAG and allow for receptor editing (16). During receptor editing, new light chain rearrangements are serially generated with remaining upstream V and J segments to pair with the existing IgH to generate a sufficiently weak BCR signal. If successful, receptor-edited immature B cells egress in the periphery as innocuous clones. If unsuccessful, their fate is cell death by apoptosis. At any one time, ∼20% of developing human B cells are undergoing secondary recombination to generate an in-frame light chain that does not render its BCR self-reactive (17).

## Inborn errors of early B cell development

Inborn errors of immunity (IEIs) result from deleterious monogenic germline variants and are characterized by defective immune cell development and/or function. Due to their compromised immune system, patients with IEIs are susceptible to recurrent and/or severe infections with a variety of pathogens (18). The important role of B cells in protection against infections and the importance of immune regulation in preventing autoimmunity and malignancy are evident from the discoveries of IEIs that disrupt B cell development and function (Table 1 and Fig. 1 E). The study of patients carrying these rare genetic defects provides unique insights into the molecular and cellular requirements for proper B cell development in humans and has greatly advanced our understanding of human B cell biology. While this review focuses on genetic variants affecting early stages of B cell development, it is important to note that numerous IEIs and common genetic variants have been identified that alter the later stages of B cell development—such as class-switching, somatic hypermutation, or germinal center reactions. These later-stage defects can similarly underlie immunodeficiency and autoimmunity. However, a comprehensive discussion of these mechanisms lies beyond the scope of this review.

**Table 1.** IEIs affecting central B cell development

Disease	Genetic defect	Inheritance	Effect	OMIM	Stage affected	Reference
AIOLOS deficiency	*IKZF3*	AD	DN	606221	Small pre-B/immature B	(19)
E47 transcription factor deficiency	*TCF3*	AD	DN	147141	CLP	(20)
E47 transcription factor deficiency	*TCF3*	AR	LOF	147141	CLP	(21)
FLT3L deficiency	*FLT3LG*	AR	LOF	600007	CLP/pro-B	(22)
Ikaros deficiency	*IKZF1*	AD	DN	-	CLP	(23)
IKAROS haploinsufficiency	*IKZF1*	AD	LOF (HI)	616873	CLP	(24)
PAX5 deficiency	*PAX5*	AR	LOF	-	Late pro-B/large pre-B	(25)
Pu.1 deficiency	*SPI1*	AD	LOF (HI)	165170	Late pro-B/large pre-B	(26)
Ataxia-telangiectasia	*ATM*	AR	LOF	607585	Early pro-B	(27)
Cernunnos/XLF deficiency	*NHEJ1*	AR	LOF	611290	Early pro-B	(28)
DCLRE1C (Artemis) deficiency	*DCLRE1C*	AR	LOF	605988	Early pro-B	(29)
DNA ligase IV deficiency	*LIG4*	AR	LOF	601837	Early pro-B	(30)
DNA PKcs deficiency	*PRKDC*	AR	LOF	600899	Early pro-B	(31)
Nijmegen breakage syndrome	*NBN*	AR	LOF	602667	Large pre-B/small pre-B	(32)
NUDCD3 deficiency	*NUDCD3*	AR	LOF	-	Early pro-B	(33)
PSMB10-associated Omenn syndrome	*PSMB10*	AD	DN*	176847	Early pro-B	(34)
RAD50 deficiency	*RAD50*	AR	LOF	613078	Late pro-B/large pre-B	(35)
RAG1 deficiency	*RAG1*	AR	LOF	179615	Early pro-B	(36)
RAG2 deficiency	*RAG2*	AR	LOF	179616	Early pro-B	(36)
Tricho-hepato-enteric syndrome due to SKIV2L mutations	*SKIV2L*	AR	LOF	614602	Early pro-B	(37)
BLNK deficiency	*BLNK*	AR	LOF	604515	Late pro-B/large pre-B	(38)
BTK deficiency, X-linked agammaglobulinemia	*BTK*	XL	LOF	300300	Late pro-B/large pre-B	(39)
Igα deficiency	*CD79A*	AR	LOF	112205	Late pro-B	(40)
Igβ deficiency	*CD79B*	AR	LOF	147245	Late pro-B	(41)
λ5 deficiency	*IGLL1*	AR	LOF	146770	Late pro-B	(42)
μ heavy chain deficiency	*IGHM*	AR	LOF	147020	Late pro-B	(43)
PIK3CD mutation (GOF) APDS1	*PIK3CD*	AD	GOF	602839	Late pro-B/large pre-B	(44)
PIK3R1 deficiency	*PIK3R1*	AR	LOF	171833	N/A	(45)
PIK3R1 deficiency (LOF) APDS2	*PIK3R1*	AD	LOF	171833	Late pro-B/large pre-B	(44)
SLC39A7 (ZIP7) deficiency	*SLC39A7*	AR	LOF	601416	Late pro-B/large pre-B	(46)
Hoffman syndrome/TOP2B deficiency	*TOP2B*	AD	DN	126431	CLP/pro-B	(47, 48)
POLE1 (Polymerase ε subunit 1) deficiency (IMAGe-1)	*POLE1*	AR	LOF	174762	N/A	(49)
POLE2 (Polymerase ε subunit 2) deficiency	*POLE2*	AR	LOF	602670	Small pre-B/immature B	(50)
Polymerase δ 1 deficiency	*POLD1*	AR	LOF	174761	Small pre-B/immature B	(51)
FNIP1 deficiency	*FNIP1*	AR	LOF	610594	Large pre-B	(52)
TRNT1 deficiency	*TRNT1*	AR	LOF	612907	Large pre-B	(53)

AR: autosomal recessive; DN: dominant negative; HI: haploinsufficient; XL: X-linked.

## Cytokines and transcription factors required for early B cell development

Early B cell development can be disrupted by IEIs affecting critical transcription factors or cytokines critical for B lineage commitment of lymphoid progenitor cells. Dominant-negative or biallelic nonsense variants in *TCF3*, encoding E12 and E47, as well as heterozygous deleterious variants in *IKZF1*, encoding IKAROS, lead to severe B cell lymphopenia (20, 21, 23, 24, 54). While TCF3 deficiency results in a loss of CLPs, *IKZF1* variants are thought to predominantly impact the differentiation trajectory of CLPs toward the B lineage, as the proportions of developing B cell subsets in IKZF1-deficient patients are largely normal. Early B cell progenitors also require adequate cytokine signaling for their further development, as highlighted in patients with FLT3L deficiency (22). Insufficient stimulation by FLT3 signaling leads to decreased proportion of B cell progenitors, likely due to insufficient skewing of differentiation toward B cells. As pro-B cells undergo further differentiation toward pre-B cells, they rely on expression of the transcription factors PAX5 (*PAX5*) and PU.1 (*SPI1*). Deficiency of either transcription factor results in the accumulation of pro-B cells in the bone marrow, which show reduced expression levels of genes required for B cell development (25, 26, 55). During the later stages of central B cell development, as the cells differentiate from pre-B cells to immature B cells, they become dependent on AIOLOS (*IKZF3*) activity. Unlike IKAROS, AIOLOS is not expressed in CLPs and early B cell progenitors, but is highly expressed in pre-B cells and subsequent developmental stages (19). Genetic defects affecting AIOLOS thus result in a block at the pre-B cell stage, also resulting in severe B cell lymphopenia (19).

## Mechanism and regulation of V(D)J recombination

V(D)J recombination is a unique process occurring in developing lymphocytes and is required for their proper development and establishment of T cell receptor (TCR) and BCR diversity (56). Severe defects of V(D)J recombination, such as complete RAG1 or RAG2 deficiency, prevent proper rearrangement and recombination of the TCR and BCR genes and thus result in severe combined immunodeficiency (SCID), which impacts both T and B cell lineages (36). Deficiency of NUDCD3 (*NUDCD3*), thought to be a chaperone of RAG1, also underlies SCID as a result of pathogenic RAG1 sequestration in nucleoli (33). RAG enzymes are critical to generate site-specific DNA double-strand breaks (DSBs) at the V, D, and J segments, requiring subsequent DSB repair through nonhomologous end joining (NHEJ). DNA-PKcs (*PRKDC*) is recruited to the open DNA ends and phosphorylation of DNA-PKcs by itself, or ATM (*ATM*) triggers an allosteric change, recruiting Artemis (*DCLRE1C*), which is critical for opening of DNA hairpins during V(D)J recombination. During the final phase of NHEJ, NHEJ1 (*NHEJ1*) interacts with the DNA ligase IV/XRCC4 (*LIG4/XRCC4*) complex to repair the broken DNA ends (56). Genetic defects affecting proteins that recognize DSBs (*PRKDC*), activate DNA-PKcs (*ATM*), cleave DNA ends (*DCLRE1C*), or are involved in DSB ligation (*NHEJ1* and *LIG4*) can all underlie radiosensitive SCID (27, 28, 29, 30, 31). Patients with deleterious variants in *RAD50* and *NBN*, both components of the nuclear MRN (MRE11, RAD50, and NBN) complex, also present with radiosensitivity due to improper capacity of their cells to repair DSBs through homologous end joining (32, 35). Low BCR diversity has been described in patients with Nijmegen breakage syndrome, caused by biallelic *NBN* variants, as well as skewed B cell development toward the pre-B cell stage (32). The report of a patient with RAD50 deficiency, presenting with bone marrow failure and B cell deficiency further, suggests a role of the MRN complex during B cell development, although MRE11-deficient patients do not typically present with signs of immunodeficiency (35). Limited V(D)J recombination was also observed in patients with PSMB10-associated Omenn syndrome (*PSMB10*) and tricho-hepato-enteric syndrome due to SKIV2L variants (*SKIV2L*), although the exact contribution of both proteins to human V(D)J recombination remains to be determined (34, 37).

## Key components of the BCR complex and signaling pathways

Following successful V(D)J recombination, a pre-BCR is expressed on large pre-B cells. The pre-BCR is composed of the two IgM heavy chains and two surrogate light chains, which associate with the Igα and Igβ signaling molecules. Genetic deficiencies affecting the constant region of the µ heavy chain (*IGHM*), the λ5 surrogate light chain (*IGLL1*), or either of the two signaling molecules (*CD79A* or *CD79B*) lead to arrested B cell development at the late pro-B cell state, which will clinically present as agammaglobulinemia (40, 41, 42, 43). A similar clinical phenotype of agammaglobulinemia or hypogammaglobulinemia is observed in patients with hemizygous variants in *BTK*, biallelic variants in *BLNK*, heterozygous gain-of-function (GOF) *PIK3CD*, or loss-of-function (LOF) *PIK3R1* variants, which all encode proteins involved in BCR signaling (38, 39, 44, 45). *PIK3CD* and *PIK3R1* encode the p85α regulatory and p110δ catalytic subunits of PI3K, respectively, which are activated following BCR activation to generate phosphatidylinositol 3,4,5-triphosphate (PIP3). PIP3 recruits BTK to the cell membrane, where it interacts with BLNK to phosphorylate specific substrates. The presence of GOF *PIK3CD* and LOF *PIK3R1* variants, which both result in enhanced PI3K activation, highlights the importance of balanced PI3K-mediated BCR signaling during B cell development (44). Notably, the presence of zinc has also been shown to be critical for proper BCR signaling during B cell development. Patients with biallelic hypomorphic variants in the zinc transporter ZIP7 (*SLC39A7*) present with a B cell differentiation block at the pro-to-pre-B cell stage (46). Hypomorphic ZIP7 reduces cytoplasmic zinc concentrations, thereby enabling increased phosphatase activity that attenuates BCR signaling in developing B cells, as shown in mouse models overexpressing variants found in patients (46).

## Factors involved in DNA replication

DNA replication and DNA repair are mechanisms that, at a first glance, appear to be essential for all human cells. However, although genetic defects alter the same cellular processes, there is considerable immunologic heterogeneity between the different genetic etiologies. T, B, and natural killer (NK) cell development is particularly sensitive to variants in genes encoding the subunits of the replicative DNA polymerases δ (POLδ) and ε (POLε), whereas variants affecting DNA topoisomerase II β (TOP2B) result in NK and B cell deficiency, with preserved T cell development (47, 49, 50, 51). *POLD1* encodes the catalytic subunit of POLδ, which is responsible for lagging-strand DNA synthesis. POLD1 deficiency mainly presents as T and NK cell lymphopenia with only two out of five reported patients presenting with B cell lymphopenia and one patient showing increased proportions of small pre-B cells (51). *POLE1* and *POLE2* encode for the catalytic and accessory subunits, respectively, of POLε. Patients with biallelic LOF *POLE1* and *POLE2* variants present with facial dysmorphism, perturbed growth, and susceptibility to infections due to immunodeficiency (49, 50). POLE1-deficient patients have been described with variable lymphocyte subset deficiencies (predominantly CD8^+^ T cells, B cells, or NK cells) or even normal lymphocyte counts (49). The only reported patient with POLE2 deficiency presented with low T and NK cell counts and an absence of mature B cells due to a developmental block at the small pre-B cell stage (50). During DNA replication and transcription, the relaxation of DNA topological stress is mediated by topoisomerases. The deficiency of TOP2B, a type II topoisomerase, underlies craniofacial and limb abnormalities, urogenital malformations, and recurrent infections (47). TOP2B-deficient patients present with low NK cell numbers and severe B cell lymphopenia, but normal T cells. Bone marrow flow cytometry of one patient revealed a complete absence of CD19^+^ B cells, affecting all stages of B cell development (48). These observations suggest that B cells are particularly sensitive to deficiencies in *POLE2* and *TOP2B*, whereas variants in *POLD1* and *POLE1* only result in a mild impairment in B cell development with variable clinical penetrance.

## Additional monogenic causes of early B cell developmental arrest

There are several monogenic causes of compromised central B cell developmental arrest in which the precise mechanism by which B cell development is altered remains elusive. Deficiency of folliculin-interacting protein 1 (*FNIP1*) has been reported in several families with skewed B cell development and increased proportions of pro- and large pre-B cells, whereas the proportions of small pre-B cells and immature B cells were reduced (52). FNIP1 modulates AMP kinase activity and was shown to be a key regulatory factor for mitochondrial function, affecting the transcription of mitochondria-associated genes (57). However, it remains unclear how perturbations in cellular energy homeostasis compromise B cell development.

B cell development is also perturbed in patients with variants in *TRNT1*, encoding the transfer RNA (tRNA) nucleotidyl transferase 1, which is essential for the synthesis of the 3′-terminal CCA sequence in cytosolic and mitochondrial tRNAs. TRNT1 deficiency was described in multiple families suffering from congenital sideroblastic anemia, immunodeficiency, periodic fevers, and developmental delay (53). Patients present with NK, T, and B cell lymphopenia associated with hypogammaglobulinemia and skewed B cell maturation in blood and bone marrow (53, 58). B cell development is impaired at the pre-B cell state, leading to an accumulation of large pre-B cell progenitors in the bone marrow and a reduction in mature B cells in the bone marrow and periphery. Bone marrow transplantation performed in one patient reverted the block in B cell maturation at 10 mo after transplant, and a second patient was reported with normalized IgG and IgA levels 2 years after receiving a bone marrow transplant, suggesting a B cell intrinsic defect in maturation (58, 59).

## Common variants affecting the human antibody repertoire

The human *IGH* locus contains 129 V, 27 D, and 9 J gene segments that are utilized during V(D)J recombination to produce a unique IgH chain of the BCR. The *IGH* locus is one of the most polymorphic and complex regions in the human genome, harboring many single nucleotide polymorphisms (SNPs), as well as large structural variants (60, 61). These structural variants include insertions, deletions, and duplications of functional genes, which result in extensive haplotype diversity and structural complexity. Studies in monozygotic twins have shown that many antibody repertoire features are correlated within twin pairs, suggesting heritability of repertoire diversity. A recent study using germline *IGH* locus long-read sequencing data showed that genetic polymorphisms and structural variants within this locus influence the antibody repertoire through impacts on gene usage frequency (62). The antibody repertoire is thereby impacted by structural variants that alter *IGH* gene copy number and SNPs and small insertions/deletions that can overlap regulatory elements and transcription factor-binding sites that might alter V(D)J recombination. Genetic variation within the *IGH* locus has also been observed across populations, involving variants that might potentiate pathogen-specific antibody production (63). Particularly high variability in the *IGH* and *IGL* loci has been observed in individuals of African descent, while individuals of European descent show higher variability in the *IGK* locus (64). These interpopulation differences might be the result of genetic drift or selection for these loci over time due to their functional consequences. It was recently shown that polymorphisms in the *IGH* genes can influence anti-influenza repertoires and functions of SARS-CoV-2–neutralizing antibodies, suggesting that the human antibody repertoire might have been subjected to the selective pressure of pathogens over time (65, 66). To date, no genome-wide or expression quantitative trait loci studies of *IGH*, *IGL*, or *IGK* usage have been undertaken, but these are likely to provide further critical insights.

## Inborn errors of central B cell tolerance

The genetic defects disturbing central B cell tolerance in humans illuminate the key pathways mediating the counterselection of autoreactive clones, namely the BCR and Toll-like receptor (TLR) signaling cascades (Table 2 and Fig. 1 E). Rare genetic alterations to BCR signaling, such as hypomorphic BTK variants dampen signal strength (39). Encumbered with less dynamic range, the normally strong BCR signals generated by self-reactive B cells are diminished and promote survival whereas the weak signals typically experienced by innocuous clones do not register at all, prompting extensive light chain rearrangement attempts. Endosomal TLR signaling pathways are also essential for mediating central tolerance, specifically purging nuclear antigen-recognizing immature B cells. There is stark enrichment of anti-nuclear naïve B cells in the blood of patients with defects in TLR signaling including IRAK-4 (*IRAK4*), MyD88 (*MYD88*), and UNC93B1 (*UNC93B1*) (72). Endosomal TLRs, which recognize danger associated molecular patterns (DAMPs) like double-stranded DNA and single-stranded RNA, co-localize with internalized complexes containing TACI (*TNFRSF13B*) and BCRs bound to nuclear antigens (71, 73, 74). The combination of strong TLR and BCR signals, which are both potentiated by TACI, is sufficient to initiate activation-induced cytidine deaminase (AID)–mediated apoptosis (75). Patients with AID deficiency or carrying heterozygous autosomal dominant (AD) *TNFRSF13B* variants are unable to tolerize anti-nuclear B cells via this mechanism and experience a host of autoantibody mediated diseases (76, 77). Interestingly, IRAK4-, MyD88-, and UNC93B1-deficient patients as well as patients with biallelic *TNFRSF13B* variants do not generally display clinical signs of autoimmunity, which emphasizes the concept that both self-antigen recognition and B cell activation are required for autoantibody production (71, 72).

**Table 2.** IEIs affecting central B cell tolerance

Disease	Genetic defect	Inheritance	Effect	OMIM	Reference
BTK deficiency, X-linked agammaglobulinemia	*BTK*	XL	LOF	300300	(39)
RAG1 deficiency	*RAG1*	AR	Hypomorphic	-	(67)
RAG deficiency	*RAG2*	AR	Hypomorphic	-	
ADA deficiency	*ADA*	AR	LOF	608958	(68)
PKCδ deficiency	*PRKCD*	AR	LOF	176977	(69)
AID deficiency	*AICDA*	AR	LOF	605257	(70)
TACI deficiency	*TNFRSF13B*	AD or AR	LOF	604907	(71)
UNC93B1 deficiency^a^	*UNC93B1*	AR	LOF	608204	(72)
IRAK4 deficiency^a^	*IRAK4*	AR	LOF	606883	
MyD88 deficiency^a^	*MYD88*	AR	LOF	602170	

AR, autosomal recessive; XL, X-linked.

a Patients with UNC93B1, IRAK4, or MyD88 deficiency do not show clinical manifestations of autoimmunity.

Hypomorphic forms of SCID can also disturb central B cell tolerance through a variety of mechanisms. For instance, adenosine deaminase (*ADA*) deficient B cells demonstrate both BCR and TLR signaling defects impairing the ability of an early B cell precursor to sense the reactivity of its BCR. Although ADA-deficient patients typically present with SCID, those with hypomorphic variants or those who are only partially treated using enzyme replacement therapy often present with autoimmunity and high levels of autoantibodies (68). Autoimmunity can also be observed in patients with hypomorphic *RAG1/2* variants, which allow for residual T and B cell development with reduced repertoire diversity (67). Hypomorphic RAG1/2 variants are likely to result in impaired central tolerance as receptor editing, which requires re-expression of RAG1/2, is disturbed and thus cannot efficiently remove autoreactive B cells from the bone marrow (67). Impaired apoptosis of autoreactive B cells was also suggested to underlie childhood-onset systemic lupus erythematosus (SLE) in patients with protein kinase C δ (*PRKCD*) deficiency (69).

## Common variants affecting central B cell tolerance

Autoimmune diseases, such as rheumatoid arthritis, SLE, and type 1 diabetes, impact ∼5% of the population. Immune repertoire analyses of B cells from human autoimmune diseases revealed a statistical increase in the numbers of polyreactive or autoreactive B cells, consistent with the possibility that these diseases are caused, in part, by defects in central B cell tolerance (78). Through genome-wide association studies (GWAS), it has become apparent that common genetic factors might predispose to multiple autoimmune disorders, with a few genetic variants being implicated in defective central tolerance or altered immune repertoire specificity (79, 80). One well-characterized variant that confers risk to develop a variety of autoimmune diseases is the c.1858C>T variant (p.R620W) in *PTPN22*, which encodes for the protein tyrosine phosphatase non-receptor type 22 (79). The *PTPN22* risk variant is associated with increased numbers of autoreactive B cells, as it alters BCR signaling, suggesting intrinsic alterations in B cell signaling can lead to defects in central tolerance. One study identified a susceptibility signal in the *IGH* locus (IGHV4-61*02) being associated with a 1.4-fold increased risk for rheumatic heart disease (80), but the structural complexity of the locus has generally limited the ability of standard GWAS approaches to identify specific causal variants. Hundreds of other loci have been associated with increased risk for various autoimmune diseases, for which the underlying mechanisms remain elusive in the majority of cases, and the discussion of which would go beyond the scope of this review (81). The incorporation of long-read sequencing (62) and other emerging genomic approaches will be useful to identify novel genetic risk factors for the development of autoantibodies specifically at the loci forming the mature BCR.

## Monogenic predisposition to B cell acute lymphoblastic leukemia (B-ALL)

Genetic variation affecting B cell development not only predisposes patients to increased susceptibility to infectious diseases or autoimmunity, but can also, in some cases, underlie increased risk for acquiring lymphoid malignancies, such as B-ALL. B-ALL is the most common type of childhood cancer and has been suspected to arise in early developing B cells, as leukemic blasts resemble pro-B cells by gene expression and other molecular features (82, 83). Genetic studies of rare leukemia-prone kindreds and large B-ALL cohorts have unraveled several genes that confer significant predisposition to B-ALL with variable clinical penetrance (Table 3). Although the precise mechanism of B-ALL predisposition remains to be determined, it is striking to observe that germline variants have been identified in genes regulating cell growth and death (*TP53* and *CDKN2A*) (84, 91), as well as in genes encoding proteins implicated in B cell development (*ETV6*, *IKZF1*, *NBN*, *TCF3*, *TRAF3IP3*, and *PAX5*) (21, 85, 86, 87, 88, 90, 92), regulation of cytokine signaling (*SH2B3*) (89), and ubiquitination (*USP9X*) (93). Genetic variation in many genes has also been reported to underlie B cell deficiency due to impaired B cell development (*IKZF1*, *NBN*, *TCF3*, and *PAX5*), suggesting shared mechanisms between altered B cell development and B-ALL predisposition (25, 32, 54, 94). It has been suggested that delayed B cell development at a stage of differentiation when B cell progenitors have high proliferative potential and high levels of RAG1/2 expression might increase the risk of acquiring deletions and translocations as a consequence of illegitimate V(D)J recombination (95, 96). Acquisition of ALL-associated deletions due to illegitimate V(D)J recombination could increase the risk of malignant transformation in developing B cells, thereby increasing the risk of acquiring B-ALL, although the underlying causality of this hypothesized mechanism remains to be shown.

**Table 3.** Genes implicated in monogenic predisposition to B-ALL

Genetic defect	Carrier status	Effect	Reference
*CDKN2A*	het	Hypomorphic/LOF	(84)
*ETV6*	het	Hypomorphic/LOF/DN	(85)
*IKZF1*	het	Hypomorphic or LOF	(86)
*NBN*	het	LOF	(87)
*PAX5*	het	LOF	(88)
*SH2B3*	hom	LOF	(89)
*TCF3*	het	LOF/HI	(90)
	hom	LOF	(21)
*TP53*	het	Hypomorphic or LOF	(91)
*TRAF3IP3*	het	Structural variant in chromosome 1q32.2	(92)
*USP9X*	het	LOF	(93)

Het, heterozygous; hom, homozygous; DN, dominant negative; HI, haploinsufficient.

## Common variants conferring increased B-ALL risk

GWAS have reproducibly identified genetic variants in at least 21 genomic loci that are associated with B-ALL risk (97, 98, 99, 100, 101, 102, 103, 104, 105, 106, 107, 108, 109) (Table 4). Although the relative risk associated with each variant is typically low (corresponding to an increase of up to twofold), cumulatively they may result in an even greater risk to B-ALL (estimated to be ∼10-fold in comparing the top and bottom deciles of risk) (109). Many of these risk loci overlap or are proximal to genes encoding for hematopoietic transcription factors, tumor suppressors, or genes involved in B cell development, including *ARID5B*, *BAK1*, *CDKN2A/CDKN2B*, *CEBPE*, *ELK3*, *ERG*, *GATA3*, *IKZF1*, *IKZF3*, *LHPP*, and *PIP4K2A* (97, 98, 99, 101, 102, 103, 104, 106, 108). Several SNPs display ancestry and B-ALL subtype-specific associations, such as variants altering *GATA3* in Ph-like B-ALL or SNPs in *IKZF1* or *ERG*, all of which have higher risk allele frequencies in individuals of Hispanic/Latino ethnicity (101, 108, 110). However, the likely causal variants have only been identified through comprehensive fine-mapping and functional experimental analyses at four ALL risk loci (*ARID5B*, *CDKN2A*, *GATA3*, and *IKFZF1*) (84, 110, 111, 112). SNPs were shown to, for example, increase *GATA3* expression, resulting in enhanced JAK-STAT signaling, or decrease *IKZF1* expression, linking B-ALL predisposition to defects in B cell lymphopoiesis (110, 112). Evolutionary analysis of the *IKZF1* rs76880433 risk allele indicated that this locus might have been under positive selection specific to the Americas, suggesting an adaptive advantage throughout human history (110). GWAS has also identified a variety of loci that were not previously linked to B cell development, but some evidence suggests that their proximal genes might be important contributors to normal B cell lymphopoiesis (104, 111).

**Table 4.** ALL risk loci identified by GWAS

Region	Nearest gene	Effect size	ALL (subtype)	Reference
5q31.1	*C5orf56*	1.29	B-ALL (high-hyperdiploidy)	(97)
6p21.31	*BAK1*	1.3	B-ALL (high-hyperdiploidy)	
17q21.32	*IGF2BP1*	1.33	B-ALL (ETV6-RUNX1 positive)	
9q21.3	*TLE1*	1.52	B-ALL	
7p12.2	*IKZF1*	1.69	B-ALL	(98)
10q21.2	*ARID5B*	1.65	B-ALL (hyperdiploid)	
14q11.2	*CEBPE*	1.34	B-ALL	
10q21.2	*ARID5B*	1.91	B-ALL	(99)
10p12.2	*PIP4K2A*	1.25	B-ALL	(100)
10p14	*GATA3*	3.85	B-ALL (Ph-like)	(101)
9p21.3	*CDKN2A*	2.99	B-ALL and T-ALL	(102)
9p21.3	*CDKN2B*	1.72	B-ALL	(103)
10q26.13	*LHPP*	1.21	B-ALL	(104)
12q23.1	*ELK3*	1.19	B-ALL	
2q22.3	*RPL6P5*	2.14	B-ALL (ETV6-RUNX1 positive)	(105)
17q21.1	*IKZF3*	1.18	B-ALL and T-ALL	(106)
7p15.3	*SP4*	1.2	B-ALL and T-ALL	
10p12.31	*BMI1*	1.27	ALL (not specified)	(107)
21q22.2	*ERG*	1.56	B-ALL	(108)
6q23	*MYB/HBS1L*	1.27	ALL (not specified)	(109)
10q21	*TET1*	1.15	ALL (not specified)	
10q21	*NRBF2*/*JMJD1C*	1.2	ALL (not specified)	

## Future outlook

The increasing availability of large-scale sequencing studies and emerging techniques like long-read sequencing are poised to enable the identification of additional genetic risk factors for susceptibility to malignancies, autoimmunity, and infectious diseases. Family-based studies and GWAS have identified rare and common genetic variants increasing disease risk to different extents. The use of rare variant association studies (RVAS) could serve as a complementary approach to identify additional genetic variants that occur at higher frequency but with lower penetrance than those observed in classical IEIs or other monogenic variants impacting B cell development (113). Variants identified through RVAS might have larger effect sizes as compared with those identified through classical GWAS and might allow for further biological insights into the risk of complex diseases, as previously shown for inherited blood cancer predisposition (114).

The study of polymorphisms within the heavy and light chain loci of the BCR through the use of long-read sequencing will allow for more comprehensive studies on the effects of germline variants on antibody repertoires in health and disease. It will be interesting to examine how BCR repertoire usage can vary across populations. Population-based studies or the use of ancient DNA samples to study selection of variants impacting B cell repertoire diversity, B cell development, or tolerance can yield fruitful insights into human genetic adaption to environmental cues, such as pathogen exposure. While a common variant might increase the risk for autoimmune/inflammatory disorders or B-ALL today, it could have conferred protection to infections in the past, such as has been suggested for variants in *TYK2*, *IKZF1*, and *FUT2* (110, 115). The investigation of the underlying mechanisms of this antagonistic pleiotropy might not only teach us more about human genetic adaptation, but might also facilitate strategies for drug development or targeted therapies with minimal risk of secondary effects.

## Conclusions

The study of genetic variation in humans has given us unique insights into B cell development in health and disease. More importantly, it has revealed shared roles for key players between seemingly distinct phenotypes, such as immunodeficiency, autoimmunity, and leukemia susceptibility, suggesting shared or overlapping mechanisms. Genetic variation has been shown to influence BCR diversity, which can result in a greater risk of infection or autoimmunity, while genetic defects in key regulators of B cell development can underlie immunodeficiency. These variants can also increase the risk of malignant transformation during B lymphopoiesis. While we structured this review based on the impact of each gene/variant on B cell development, we suggest that each variant should be viewed from a more holistic perspective and not through the phenotypic lens typically applied by immunologists, rheumatologists, infectious disease specialists, hematologists, or oncologists. Although each discipline provides a unique perspective on disease pathogenesis, the widespread role of B cell development in health and disease emphasizes a global role for many genetic variants impacting this process in seemingly disparate processes ranging from infectious susceptibility to cancer risk.
